# Blood microRNA 202-3p associates with the risk of essential hypertension by targeting soluble ST2

**DOI:** 10.1042/BSR20200378

**Published:** 2020-05-05

**Authors:** Lu Li, Danrong Zhong, Yudan Xie, Xinlei Yang, Zuozhong Yu, Dangui Zhang, Xinghua Jiang, Yanqing Wu, Fangqin Wu

**Affiliations:** 1 Research Center of Translational Medicine, The Second Affiliated Hospital of Shantou University Medical College, Guangdong, China; 2 Department of Cardiovascular Medicine, The Second Affiliated Hospital of Nanchang University, Jiangxi, China; 3 Department of Cardiovascular Medicine, The Second Affiliated Hospital of Shantou University Medical College, Guangdong, China; 4 Biobank Center, The Second Afflicted Hospital of Nanchang University, Jiangxi, China; 5 Center for Pathgen Biology and Immunology, Shantou University Medical College, Guangdong, China

**Keywords:** essential hypertension, miR-202-3p, sST2

## Abstract

MicroRNA (miR)-202-3p has attracted a great deal of attention in the fields of oncology, gynecology, and metabolic disorders. However, its role in cardiovascular diseases remains to be clarified. We previously found that disruption of miR-202-3p mediated regulation of expression of soluble (s)ST2, a decoy receptor for interleukin (IL)-33, promotes essential hypertension (EH). In the present study, we first measured miR-202-3p expression levels in the blood of 182 EH cases and 159 healthy controls using TaqMan assays. miR-202-3p levels were shown to be significantly higher in EH cases than controls (fold change = 3.58, *P*<0.001). Logistic regression analysis revealed that higher miR-202-3p expression was associated with an increased occurrence of EH (adjusted odds ratio (OR): 1.57; 95% confidence interval (CI), 1.36–1.82; *P*<0.001). Addition of miR-202-3p to traditional risk factors showed an additive prediction value for EH. Further functional experiments indicated that miR-202-3p could be induced by angiotensin II (Ang II) and inhibited by Ang II-triggered soluble ST2 (sST2) expression in a negative feedback manner. Moreover, blood miR-202-3p levels were negatively correlated with sST2 expression *in vivo*. Our study shows that blood miR-202-3p levels were significantly associated with the occurrence of EH. These findings indicate that miR-202-3p exerts a protective role against EH by antagonizing the induction of sST2 by Ang II.

## Introduction

Hypertension is a chronic disease characterized by a continuous increase in arterial blood pressure. It is also a critical risk factor for mortality and morbidity in several cardiovascular diseases including stroke, heart failure, and kidney failure [1]. According to a World Health Organization report of 2017, 23.2% of Chinese adults suffer from hypertension, so ∼245 million individuals are affected in China [2]. Essential hypertension (EH), as the most common disease type, accounts for ∼95% of cases. Hypertension pathogenesis is a multifactorial process involving the interaction of genetic and environmental factors. Besides traditional risk factors such as a high-salt diet, smoking, alcohol, and obesity, 30–50% of blood pressure variations can be attributed to genetic factors.

microRNAs (miRNAs) are a class of endogenous non-coding RNAs of 22 nucleotides in length that are involved in the post-transcriptional regulation of target genes by interacting with the 3′ untranslated region (3′ UTR) of mRNAs. As epigenetic factors, miRNAs have received increasing attention in the occurrence and progression of diseases. Moreover, in recent decades, several population-based research and animal model studies have been conducted to explore the differential expression profiles of hypertension-associated miRNAs. Their findings suggested that miRNAs are not only involved in multiple key steps of the onset and development of EH [3–5], but that they could also be used to predict disease risk and severity as novel biomarkers [6–8].

Peripheral blood contains large numbers of white blood cells, platelets, and red blood cells. Previous studies have suggested that changes in the counts, species, and intracellular gene expression profiles of leukocytes are closely related to hypertension development [9,10]. Therefore, work has focused on the association between blood cell miRNAs and EH occurrence as well as underlying biological mechanisms. Torres-Paz et al. showed that the up-regulation of miR-33a-5p expression in monocytes was significantly positively correlated with carotid intima thickness and increased the risk of hypertension in a Mexican population [11]. Additionally, Yin et al. reported that the expression of miR-128 in peripheral blood was gradually up-regulated during hypertension progression, which aggravated myocardial injury through inhibiting c-Met [12]. Moreover, Parthenakis et al. found that miR-208b and miR-133a were significantly positively correlated with urinary albumin excretion levels in newly diagnosed hypertension patients, suggesting that they could serve as a new generation of biomarkers for improved monitoring of end organ damage in hypertension [13]. Therefore, we proposed that the identification of EH-associated miRNA in peripheral blood cells and the exploration of underlying mechanisms could provide new perspectives in the diagnosis, treatment, and prevention of this disease.

Emerging evidence suggests that miR-202-3p could function as a suppressor for a series of tumors, including osteosarcoma, colorectal cancer, gastric cancer, cervical cancer, and thyroid cancer [14,15]. By controlling a variety of target genes such as cyclin D1, γ-catenin, and bcl-2, miR-202-3p plays an essential role in multiple physiological and pathogenic processes of tumor progression including cell proliferation, invasion, and apoptosis. It has also been reported to be involved in various metabolic and cardiovascular diseases. For example, Fornari et al. found that miR-202-3p regulated Ccr7 and cd247, interfered with immune homeostasis, and led to the occurrence of type 1 diabetes [16]. Recently, Wu et al. showed that miR-202-3p overexpression alleviated myocardial ischemia and reperfusion injury by activating the transforming growth factor β-1/Smads pathway and protecting against myocardial infarction and fibrosis [17].

In 2017, we conducted a case–control study to explore the association between genetic variants in the interleukin (IL)-33/ST2 pathway and the risk of hypertension and found that a common polymorphism within the 3′ UTR of the ST2 gene interrupted the regulation of soluble (s)ST2 expression by miR-202-3p and promoted the occurrence of EH [18]. Because the IL-33 decoy receptor soluble ST2 (sST2) is widely recognized as a risk factor for hypertension [19,20], we hypothesized that the inhibition of sST2 by miR-202-3p was critical for the onset and development of EH. However, the etiological and pathological role of miR-202-3p in EH remained unclear. In the present study, we first conducted a case–control study to validate the association between blood cell levels of miR-202-3p and the occurrence of EH. Then, to investigate the pathological role of miR-202-3p in EH, we analyzed the effects of miR-202-3p on sST2 expression both *in vitro* and *in vivo*.

## Methods

### Study design and population

The 182 EH cases for miRNA detection were consecutively recruited from the Second Affiliated Hospital of Nanchang University in Nanchang City, Jiangxi province, China between November 2016 and August 2017. Hypertension was diagnosed as average SBP ≥ 140 mmHg, and/or average diastolic blood pressure (DBP) ≥ 90 mmHg, and/or self-reported current treatment for hypertension with antihypertensive drugs. A total of 159 control subjects were from the same community as the patients. Control subjects showed SBP < 140 mmHg, DBP < 90 mmHg, and had never been treated for hypertension. Blood pressure was measured according to reported guidelines [21]. People participating in the physical examination at the Second Affiliated Hospital of Nanchang University were recruited and frequency matched by age and sex with patients, with determined to be free of EH and peripheral atherosclerotic arterial disease by medical history and clinical examinations. Five milliliters of fresh vein blood samples were collected from each participant with EDTA-anticoagulant tubes. The plasma and blood cells were separated by centrifugation at 2000×***g*** for 10 min at 4°C, and then stored at −80°C until use. The questionnaires were used to collect general characteristics and results of clinical biochemistry tests for all participants by trained interviewers. The study protocol conformed to the ethical guidelines of the 1975 Declaration of Helsinki and was approved by the ethics committee of the Second Affiliated Hospital of Nanchang University, and all participants provided written informed consent.

### Total RNA isolation

For miRNA detection in blood samples, approximately 200 μl of blood was used to extract total RNAs using the mirVana PARIS miRNA Isolation Kit (Ambion1556) following the manufacturer’s protocol. For gene expression detection in cultured cells, total RNAs were isolated using TRIzol reagent (Invitrogen) following the manufacturer’s instructions.

### qRT-PCR assays

miRNA expression was detected as previously described [22]. In brief, input RNAs were reverse transcribed (RT) using the TaqMan miRNA Reverse Transcription Kit (Applied BioSystems, Foster City, CA) following the manufacturer’s protocol. RT products were diluted 1:5 and subjected to qPCR in triplicate using the TaqMan miRNA Assay Kit (Applied BioSystems, Foster City, CA) according to the manufacturer’s protocol. miRNA expression levels were normalized to U6b and calculated using the equation 2^−Δ*C*_t_^, where Δ*C*_t_ = cycle threshold (*C*_t_) (miRNA) − *C*_t_ (U6b).

For gene expression analysis, first strand cDNA was obtained using SuperS High Capacity cDNA Reverse Transcription Kit (Applied BioSystems). Quantitative PCR was conducted to detect gene expressions using PowerUp™ SYBR™ Green Master Mix (Applied BioSystems). The mRNA levels were normalized to β-actin mRNA levels. The primers for the genes were as follows: β-actin (F: 5′-CCTGGCACCCAGCACAAT-3′, R: 5′-GCCGATC CACACGGAGTACT-3′); and sST2 (F: 5′-GGCACACCGTAAGAC TAAGTAG-3′; R: 5′-CAATTTAAGCAGCAGAGAAGCTCC-3′).

### Chemicals, cell culture and transfection

Human acute monocytic leukemia cell line THP-1 cells, purchased from the type culture collection of the Chinese Academy of Sciences (Shanghai, China), were maintained in RPMI-1640 medium (Gibco) supplemented with 10% fetal bovine serum (FBS), sodium pyruvate and GlutaMax. Angiotensin II (Ang II) was purchase from Sigma–Aldrich Chemical Reagent Co., Ltd (St. Louis, Missouri, U.S.A.). miRNA mimics and inhibitor were synthesized by Ruibo Bio Co., Ltd. (Guangzhou, China) and transfected into cells alone or with the reporter plasmids using Lipofectamine 3000 (Invitrogen) in accordance with the manufacturer’s protocol. All experiments were conducted with cells at a logarithmic stage of growth curve.

### Biological variable determination

The levels of biological variables such as total cholesterol (TC), triglyceride (TG), and fasting blood glucose (FBG) etc. were measured by standard laboratory procedures at the Department of Clinical Laboratory, The Second Affiliated Hospital of Nanchang University.

### Statistical analysis

The general characteristics of cases and controls are presented as mean ± standard deviation (SD), median with interquartile range (IQR) or number with percentage. Comparison of the differences was conducted by Student’s *t* test, Mann–Whitney U test, or the Chi-square test depending on the distribution of data. The difference in circulating miR-202-3p levels between cases and controls was examined by Student’s *t* test. The odds ratios (ORs) and 95% confidence intervals (CIs) were calculated to indicate the association of miR-202-3p expression with the occurrence of EH by unconditional logistic regression model, with an adjustment of traditional risk factors including age, sex, smoking, drinking, TC, fasting glucose (FG), and family history of hypertension. The ability for miR-202-3p to discriminate EH cases from controls was analyzed by receiver operating characteristic (ROC) curves and reclassification analysis in two models. The baseline model was composed of traditional risk factors, and the extended model incorporated miR-202-3p expression with traditional risk factors. Correlations between miRNA expression and clinical parameters were analyzed by the Pearson or Spearman correlation tests. All statistical analyses were performed using SPSS 12.0 software (Statistical Package for the Social Sciences, Chicago, IL, U.S.A.). A value of *P*<0.05 was considered significant (two-tailed).

## Results

### General characteristics of the study population

General characteristics of the population are shown in Table 1. Sex ratios of case and control groups were similar, but the age (59–72 years), creatinine (65.81, 101.23), homocysteine (11.64, 18.35), uric acid (300.51, 481.51), body mass index (BMI, 25.14 ± 3.64), and alcohol consumption (20.2%) of EH patients were significantly higher than the healthy control group (*P*<0.05). In contrast, the levels of TC (4.42 ± 1.17 mmol/l), high-density lipoprotein cholesterol (HDL-c; 1.05 ± 0.27 mmol/l), low-density lipoprotein cholesterol (LDL-c; 2.81 ± 1.05 mmol/l), and a history of hypertension (18.5%) were significantly lower than those in the control group (*P*<0.05), possibly because of the application of cholesterol-lowering medications in this population. No significant differences were found for the other variables between the two groups.

**Table 1:** General characteristics of the study population

Variables	Control (*n*=159)	EH (*n*=182)	*P-value*
Age, years	60 (50, 67)	64 (59, 72)	0.001^1^
Male, %	55.3	52.2	0.561^2^
FG, mmol/l	5.12 (4.80, 5.63)	5.19 (4.77, 5.90)	0.376^1^
TC, mmol/l	5.24 ± 0.98	4.42 ± 1.17	<0.001^3^
TG, mmol/l	1.26 (0.91, 1.84)	1.25 (1.00, 1.73)	0.596^1^
HDL-c, mmol/l	1.25 ± 0.29	1.05 ± 0.27	<0.001^3^
LDL-c, mmol/l	3.31 ± 0.84	2.81 ± 1.05	<0.001^3^
Creatinine	69.35 (59.60, 80.11)	79.28 (65.81, 101.23)	<0.001^1^
Homocysteine	12.17 (10.66, 15)	13.86 (11.64, 18.35)	0.002^1^
Uric acid	323.20 (282.73, 395.72)	381.94 (300.51, 481.51)	<0.001^1^
PLT (×10^9^/l)	212.92 ± 55.18	205.74 ± 66.01	0.284^3^
PDW, %	13.4 (11.8, 15.3)	12.8 (11.5, 14.88)	0.243^1^
MPV, fl	11.00 (10.3, 11.9)	10.90 (10.23, 11.80)	0.624^1^
PCT	0.24 ± 0.05	0.23 ± 0.06	0.124^3^
BMI	23.58 ± 3.06	25.14 ± 3.64	<0.001^3^
miR-202-3p	−1.71 ± 2.27	0.13 ± 1.94	<0.001^3^
Smoking, %	22.3	18.5	0.394^2^
Drinking, %	8.9	20.2	0.004^2^
Family history of hypertension, %	29.9	18.5	0.015^2^

Data are expressed as mean ± SD, median (25th, 75th quartiles) or percentages.

Abbreviations: MPV, mean platelet volume; PCT, plateletcrit; PDW, platelet distribution width; PLT, blood platelet count.

1 Mann–Whitney U test for the differences between EH patients and controls.

2 Chi-square test for the difference in the distribution frequencies between EH patients and controls.

3 Student’s *t* test for the difference between EH patients and controls.

### Association between miR-202-3p blood levels and EH

**Figure 1:**
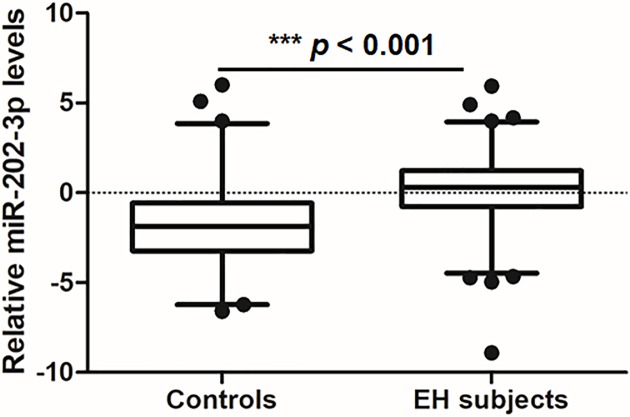
Expression levels of miR-202-3p in EH patients and control subjects The expression of miR-202-3p was detected in 159 patients and 168 control subjects. Relative expression levels were normalized to U6b and then log 2-transformed. The whiskers of the plots represent the 2.5–97.5 percentiles. ***, *P*<0.001, miR-202-3p expression in EH patients vs. controls.

As shown in Figure 1, the level of miR-202-3p in peripheral blood cells was significantly higher in EH patients than in controls (log-transformed expression levels relative to U6b, 0.13 ± 1.94 vs. –1.71 ± 2.27; *P*<0.001). Logistic regression analysis indicated that higher levels of miR-202-3p were associated with an increased risk of EH (adjusted OR: 1.57; 95% CI: 1.36−1.82; *P*<0.001; Table 2) after adjusting for age, sex, smoking, drinking, BMI, TC, FG, and a history of hypertension.

**Table 2:** Association of the expression level of miR-202-3p with EH

Variables	OR (95% CI)^1^	*P-value*
Age	1.04 (1.01–1.07)	0.007
Male	0.54 (0.27–1.08)	0.083
Smoking	1.20 (0.53–2.71)	0.669
Drinking	0.40 (0.16–1.01)	0.053
BMI	1.20 (1.10–1.31)	<0.001
TC	0.48 (0.36–0.64)	<0.001
FG	1.14 (0.94–1.40)	0.192
Family history of hypertension, %	1.59 (0.81–3.10)	0.176
miR-202-3p	1.57 (1.36–1.82)	<0.001

1 By logistic regression analysis adjusted with age, sex, smoking, drinking, BMI, TC, FG, and family history of hypertension.

### Diagnostic value of miR-202-3p

As shown in Table 3, the area under the ROC curve was similar between the baseline model (0.77; 95% CI, 0.71–0.82; *P*<0.001) and the combined miR-202-3p prediction model (0.84; 95% CI: 0.79–0.88; *P*<0.001). The net reclassification improvement (NRI) and integrated discrimination improvement (IDI) were calculated to determine the ability of miRNA to reclassify patients misclassified by the baseline model. The results showed that miR-202-3p was able to reclassify a significant portion of patients with an NRI of 94.3% (95% CI: 75.0–113.5; *P*<0.001) and an IDI of 0.131 (95% CI: 0.094–0.167; *P*<0.001).

**Table 3:** AUC, NRI, and IDI calculations for miR-202-3p

Variables	miR-202-3p	*P*-value
Baseline model AUC (95% CI)	0.77 (0.71, 0.82)	<0.0001
Extended model AUC (95% CI)	0.84 (0.79, 0.88)	<0.0001
NRI, % (95% CI)	94.3 (75.0, 113.5)	<0.0001
IDI, (95% CI)	0.131 (0.094, 0.167)	<0.0001

AUC calculations are based on a multivariate logistic regression analysis including age, sex, smoking, drinking, diabetes mellitus, TG and family history of hypertension (baseline model) or the addition of miR-202-3p (extended model). Abbreviation: AUC, area under the ROC curve.

### Correlation between miR-202-3p expression and clinical variables

Conventional risk factors including smoking, drinking, and a history of hypertension contribute to the occurrence of EH. Therefore, we next analyzed the correlation between miR-202-3p and various clinical variables. As shown in Table 4, blood levels of miR-202-3p were significantly positively correlated with male sex (r = 0.114, *P*=0.036), drinking (r = 0.115, *P*=0.036), and BMI (r = 0.14, *P*=0.011). It is also widely accepted that lipid and glucose metabolism play critical roles in the development of EH. Our findings suggested that blood miR-202-3p levels were significantly negatively correlated with those of TC (r = −0.127, *P*=0.021) and HDL-c (r = −0.15, *P*=0.006). Moreover, miR-202-3p was significantly positively correlated with creatinine (r = 0.115, *P*=0.034), uric acid (r = 0.115, *P*=0.034), and homocysteine (r = 0.186, *P*=0.006) and negatively correlated with platelet count in EH patients (r = −0.108, *P*=0.048). No significant correlations were found with other variables.

**Table 4:** Correlation analysis between miR-202-3p expression and clinical variables in combined population

Variables	*r*	*P-value*
Age	0.002	0.971^1^
Sex	0.114	0.036^1^
Smoking	−0.06	0.27^1^
Drinking	0.115	0.036^1^
Family history of hypertension %	−0.011	0.846^1^
TG	0.055	0.322^1^
TC	−0.127	0.021^2^
HDL-c	−0.15	0.006^2^
LDL-c	−0.09	0.104^2^
FG	0.038	0.495^1^
BMI	0.14	0.011^2^
Creatinine	0.115	0.034^1^
Uric acid	0.115	0.034^1^
Homocysteine	0.186	0.006^1^
MPV	0.09	0.101^1^
PCT	−0.095	0.084^2^
PDW	0.078	0.159^1^
PLT	−0.108	0.048^2^

Abbreviations: MPV, mean platelet volume; PCT, plateletcrit; PDW, platelet distribution width; PLT, blood platelet.

1 Spearman correlation test.

2 Pearson correlation test.

### miR-202-3p inhibits sST2 expression induced by Ang II

**Figure 2:**
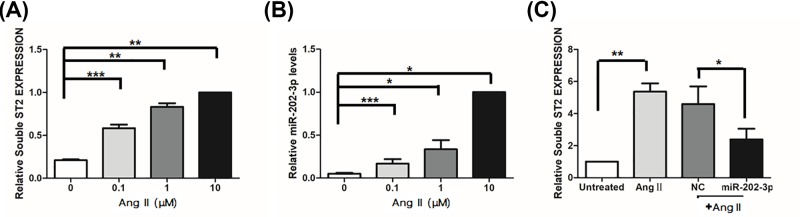
miR-202-3p inhibits sST2 expression induced by Ang II Human macrophage THP-1 cells were seeded into six-well plate and starved overnight. After that, cells were treated with different concentrations of Ang II (0, 0.1, 1 and 10 μM) for 24 h, respectively. After that, cells were harvested to analyze the expression of sST2 (**A**) and miR-202-3p (**B**). The mRNA levels were normalized to β-ACTIN mRNA and the levels of miRNA were normalized to U6b. All data were expressed as mean ± SD and the differences of gene expression between groups treated with different doses of ang II were examined by Student’s *t* test. *P<0.05, **P<0.01, ***P<0.005 vs untreated control. (**C**) THP-1 cells were seeded into six-well plate and transfected with miR-202-3p mimic or negative control mimic (NC). At 24 h post transfected, cells were further stimulated with 10 μM Ang II for another 24 h. After that, cells were collected to analyze the expression of sST2. The difference of gene expression between groups transfected with miR-202-3p and negative control was examined by Student’s *t* test. **P<0.01 vs untreated control, *P<0.05 vs negative control mimic. All data represent three independent experiments.

It is widely accepted that vascular inflammation contributes greatly to the pathogenesis of EH [23]. To reveal the underlying mechanism by which miR-202-3p is involved in EH pathogenesis, we applied the prohypertensive reagent Ang II to human THP-1 macrophage cells to induce an inflammatory response. As shown in Figure 2B, the endogenous expression of miR-202-3p in THP-1 cells was gradually up-regulated by Ang II in a dose-dependent manner. Because we previously found that sST2 was controlled by miR-202-3p [18]. We next examined the effects of miR-202-3p on sST2 expression in the Ang II-induced monocytic inflammatory response. Figure 2A shows that the expression of sST2 increased sharply after Ang II stimulation, and that this could be abrogated by miR-202-3p transfection (Figure 2C).

### Blood cell levels of miR-202-3p were negatively correlated with sST2 expression in PBMCs

**Figure 3:**
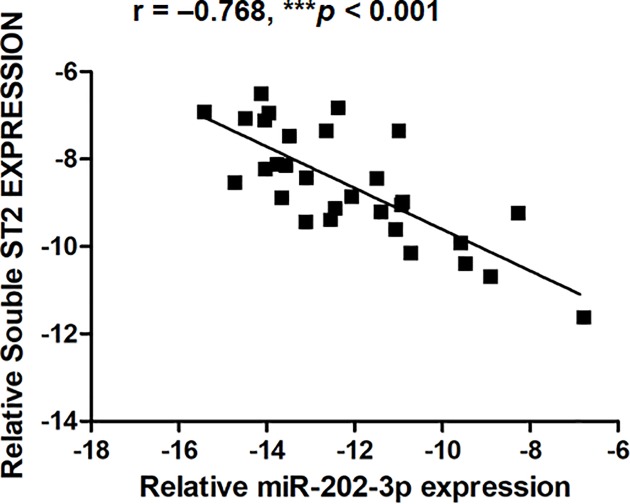
Blood levels of miR-202-3p were negatively correlated with sST2 expression in PBMCs Human fresh vein blood samples from 30 healthy controls were collected and centrifuged to remove plasma. Then, the remnants were mixed with TRIzol LS reagent immediately. Total RNA was extracted to monitor the levels of miR-202-3p and sST2 expression by Quantitative PCR. The correlation of miR-202-3p levels with sST2 expression were examined by Pearson correlation test.

To validate the regulation of sST2 by miR-202-3p *in vivo*, we examined the correlation between blood levels of miR-202-3p and sST2 expression in PBMCs from 30 healthy controls. As shown in Figure 3, miR-202-3p levels were significantly negatively correlated with the expression of sST2 (r = –0.768, *P*<0.001).

## Discussion

In the present study, we proposed a negative feedback mechanism mediated by miR-202-3p against EH and obtained the following supporting evidence. First, our large sample case–control study found that higher miR-202-3p levels of blood cells were associated with an increased occurrence of EH. ROC curve and reclassification analysis indicated that the combination of miR-202-3p with traditional risk factors improved the predictive value of EH risk. Then, we showed that the expression of both sST2 and miR-202-3p could be induced by Ang II in a dose-dependent manner and that miR-202-3p overexpression inhibited the up-regulation of sST2 triggered by Ang II. Further, an *in vivo* assay suggested that miR-202-3p levels were negatively correlated with sST2 expression in the peripheral blood cells of 30 healthy controls. Collectively, our results suggest that miR-202-3p is involved in the development of EH partially by down-regulating sST2 expression via a feedback manner.

Systemic and local inflammation are both known to play an important role in the pathogenesis of EH. IL-33 is a newly discovered member of the IL-1 family that induces a Th2-type immune response by interacting with the ST2 receptor on cell membranes [24]. The ST2 gene encodes four isomers: sST2, ST2L, ST2V, and ST2LV, of which sST2 acts as a decoy receptor for IL-33, blocking the IL-33/ST2 signaling pathway by binding to IL-33, and inhibiting Th2-type inflammatory immune responses [25,26]. IL-1RAcP, another gene in this pathway, determines the affinity between IL-33 and ST2 [27]. In recent years, multiple pieces of evidence have shown that the IL-33/ST2 pathway has a crucial function in the pathogenesis of EH. The relationship between sST2 and hypertension has attracted particular attention. Ho et al. found that increased plasma concentrations of sST2 were significantly associated with EH risk [28], while Yin et al. suggested that sST2 plasma levels could be used as a biomarker for EH risk prediction [29].

In the present study, we revealed that blood cell levels of miR-202-3p were significantly increased in EH patients compared with controls and that higher levels were associated with an increased risk of EH, and addition of miR-202-3P to the traditional risk factor model increased the prediction probability. Further functional analysis suggested an underlying mechanism involving the exertion of anti-hypertensive effects by miR-202-3p partially through the suppression of sST2 in vascular inflammation. This is in-line with our previous study that indicated that the disruption of sST2 expression regulation by miR-202-3p contributed significantly to EH development [18]. To the best of our knowledge, this study documented a significant risk predicting value of miR-202-3p for the occurrence of EH for the first time. Although past studies have focused on the anti-tumor role of miR-202-3p, our study and others’ suggested that miR-202-3p played a critical role in the development of diverse cardiovascular diseases [17,18].

Gene annotation enrichment analysis for potential targets of miR-202-3p suggested that it might be implicated in cardiovascular and metabolic diseases (data not shown). We also conducted a population-based study and cell experiments to determine the etiological role of miR-202-3p in EH development. Our findings suggested that higher blood levels of miR-202-3p associated with an increased risk of EH and that sST2 is a critical target for miR-202-3p to exert anti-hypertensive effects. However, sST2 might not be the only target of miR-202-3p in EH pathogenesis. For example, the target genes CCR7 and CD247 were shown to be involved in the regulation of immune inflammatory cell function, contributing to end organ damage in hypertension [30,31]. Moreover, TRPM6, an identified target of miR-202-3p, encodes a transient receptor potential ion channel that plays an important role in the regulation of vascular tone and blood pressure [32,17], while MALAT-1, a long noncoding RNA targeted by miR-202-3p, was involved in vascular remodeling and promoted hypertension [33,34]. To the best of our knowledge, this study is the first to illustrate a mechanistic link between miR-202-3p and EH. However, additional efforts should be made to comprehensively reveal the mechanism by which miR-202-3p influences EH development.

As we all know, high plasma level of uric acid and homocysteine both contribute greatly to EH pathogenesis [35,36]. Notably, we found miR-202-3p expression was positively correlated with uric acid and homocysteine (Table 4), inconsistent with that higher expression of miR-202-3p could be detected in EH patients (Figure 1). Wainwright et al. have indicated that miR-202-3p transcription was directly controlled by a transcription factor SOX9 [37]. Hyperhomocysteinemia and hyperuricemia both could cause an induction of SOX9 expression [38,39]. Thus, we suggest the SOX9-miR-202-3p-sST2 signaling axis might be critical for the development of hypertension complicated by hyperuricemia and hyperhomocysteinemia and our study will focus on it in next step.

miR-202-3p regulates the proliferation, apoptosis, and function of Sertoli cells and plays a critical role in spermatogenesis [40]. Higher expression of miR-202-3p could be detected in males. We speculated the levels of miR-202-3p might affect the incidence of hypertension. Thus, the expressions of miR-202-3p in PBMCs in our population were examined. We failed to detect higher expression of miR-202-3p in both male patients and total males, probably because of small sample size. Even in the controls group, the levels of miR-202-3p in peripheral blood cells were significantly higher in females than in males (log-transformed expression levels relative to U6B, −1.10 ± 2.33 vs. –2.20 ± 2.10, *P*=0.002).

The present study has a number of limitations. First, miRNAs often exert their effects synergistically. Thus, systematically screening all IL-33/ST2 pathway-targeting miRNAs could help determine the pathomechanism of EH and improve the prediction efficiency. Second, the effect of miR-202-3p in the progression of EH should be evaluated in future clinical studies. Finally, the biological consequence of miR-202-3p depletion on blood pressure variation and end organ damage should be monitored using spontaneous hypertensive rat models.

In summary, our study showed that blood levels of miR-202-3p increased in EH patients and were associated with EH. Further functional analyses suggested that miR-202-3p suppressed sST2 expression in a feedback manner both *in vitro* and *in vivo*. Thus, miR-202-3p appears to exert an anti-hypertensive role but more efforts are required to reveal the underlying mechanisms.

## Abbreviations

Ang II: angiotensin II
bcl-2: B-cell lymphoma-2
BMI: body mass index
CI: confidence interval
*C*_t_: cycle threshold
DBP: diastolic blood pressure
EH: essential hypertension
FG: fasting glucose
HDL-c: high-density lipoprotein cholesterol
IDI: integrated discrimination improvement
IL: interleukin
IL-1RAcP: interleukin-1 receptor accessory protein
IQR: interquartile range
MALAT-1: metastasis-associated lung adenocarcinoma transcript 1
miR: microRNA
NRI: net reclassification improvement
OR: odds ratio
PBMC: peripheral blood mononuclear cell
qRT-PCR: real-time quantitative reverse transcription polymerase chain reaction
ROC: receiver operating characteristic
SBP: systolic blood pressure
SOX9: SRY-box transcription factor 9
Sst2: soluble ST2
ST2: suppression of tumorigenicity 2
TC: total cholesterol
TRPM6: potential cation channel subfamily M member 6
3′ UTR: 3′ untranslated region

## References

[1] (2007) A new weapon against hypertension. Recently approved medication lowers blood pressure, reducing risks of heart failure, stroke, heart attack, aneurysm, and kidney failure. Heart Advis. 10, 3 17654793

[2] Butler M.G. (2010) Genetics of hypertension. Current status. J. Med. Liban. 58, 175–178 21462849 PMC5132177

[3] Li X., Wei Y. and Wang Z. (2018) microRNA-21 and hypertension. Hypertens. Res. 41, 649–661 10.1038/s41440-018-0071-z 29973661

[4] Nemecz M., Alexandru N., Tanko G. and Georgescu A. (2016) Role of microRNA in endothelial dysfunction and hypertension. Curr. Hypertens. Rep. 18, 87 10.1007/s11906-016-0696-8 27837398 PMC7102349

[5] Miao C., Chang J. and Zhang G. (2018) Recent research progress of microRNAs in hypertension pathogenesis, with a focus on the roles of miRNAs in pulmonary arterial hypertension. Mol. Biol. Rep. 45, 2883–2896 10.1007/s11033-018-4335-0 30298350

[6] Xu J., Zhao J., Evan G., Xiao C., Cheng Y. and Xiao J. (2012) Circulating microRNAs: novel biomarkers for cardiovascular diseases. J. Mol. Med. (Berl.) 90, 865–875 10.1007/s00109-011-0840-5 22159451

[7] Perez-Hernandez J., Olivares D., Forner M.J., Ortega A., Solaz E., Martinez F. et al. (2018) Urinary exosome miR-146a is a potential marker of albuminuria in essential hypertension. J. Transl. Med. 16, 228 10.1186/s12967-018-1604-6 30107841 PMC6092786

[8] Huang Y., Tang S., Huang C., Chen J., Li J., Cai A. et al. (2017) Circulating miRNA29 family expression levels in patients with essential hypertension as potential markers for left ventricular hypertrophy. Clin. Exp. Hypertens. 39, 119–125 10.1080/10641963.2016.1226889 28287884

[9] Sun Y.T., Gong Y., Zhu R., Liu X., Zhu Y., Wang Y. et al. (2015) Relationship between white blood cells and hypertension in Chinese adults: the Cardiometabolic Risk in Chinese (CRC) study. Clin. Exp. Hypertens. 37, 594–598 10.3109/10641963.2015.1036058 26114355

[10] Huan T., Esko T., Peters M.J., Pilling L.C., Schramm K., Schurmann C. et al. (2015) A meta-analysis of gene expression signatures of blood pressure and hypertension. PLoS Genet. 211, e1005035 10.1371/journal.pgen.1005035 PMC4365001 25785607

[11] Torres-Paz Y.E., Huesca-Gómez C., Sánchez-Muñoz F., Martínez-Alvarado R., Soto M.E., Torres-Tamayo M. et al. (2018) Increased expression of miR-33a in monocytes from Mexican hypertensive patients in elevated carotid intima-media thickness. J. Hum. Hypertens. 32, 681–690 10.1038/s41371-018-0102-x 30232400

[12] Yin J., Liu H., Huan L., Song S., Han L., Ren F. et al. (2017) Role of miR-128 in hypertension-induced myocardial injury. Exp. Ther. Med. 14, 2751–2756 10.3892/etm.2017.4886 28928797 PMC5590046

[13] Parthenakis F.I., Marketou M.E., Kontaraki J.E., Maragoudakis F., Maragkoudakis S., Nakou H. et al. (2016) Comparative microRNA profiling in relation to urinary albumin excretion in newly diagnosed hypertensive patients. J. Hum. Hypertens. 30, 685–689 10.1038/jhh.2016.15 26984682

[14] Chen J., Yin J., Liu J., Zhu R.X., Zheng Y. and Wang X.L. (2019) MiR-202-3p functions as a tumor suppressor and reduces cell migration and invasion in papillary thyroid carcinoma. Eur. Rev. Med. Pharmacol. Sci. 23, 1145–1150 30779083 10.26355/eurrev_201902_17005

[15] Wang Q., Huang Z., Guo W., Ni S., Xiao X., Wang L. et al. (2014) microRNA-202-3p inhibits cell proliferation by targeting ADP-ribosylation factor-like 5A in human colorectal carcinoma. Clin. Cancer Res. 20, 1146–1157 10.1158/1078-0432.CCR-13-1023 24327274

[16] Fornari T.A., Donate P.B., Assis A.F., Macedo C., Sakamoto-Hojo E.T., Donadi E.A. et al. (2015) Comprehensive survey of miRNA-mRNA interactions reveals that Ccr7 and Cd247 (CD3 zeta) are posttranscriptionally controlled in pancreas infiltrating T lymphocytes of non-obese diabetic (NOD) mice. PLoS ONE 10, e0142688 10.1371/journal.pone.0142688 26606254 PMC4659659

[17] Wu H.Y., Wu J.L. and Ni Z.L. (2019) Overexpression of microRNA-202-3p protects against myocardial ischemia-reperfusion injury. activation of TGF-β1/Smads signaling pathway by targeting TRPM6. Cell Cycle 18, 621–637 10.1080/15384101.2019.1580494 30810438 PMC6464590

[18] Wu F., Li L., Wen Q., Yang J., Chen Z., Wu P. et al. (2017) A functional variant in ST2 gene is associated with risk of hypertension via interfering MiR-202-3p. J. Cell. Mol. Med. 21, 1292–1299 10.1111/jcmm.13058 28121058 PMC5487927

[19] Farcaş A.D., Anton F.P., Goidescu C.M., Gavrilă I.L., Vida-Simiti L.A. and Stoia M.A. (2017) Serum soluble ST2 and diastolic dysfunction in hypertensive patients. Dis. Markers 2017, 2714095 10.1155/2017/2714095 28566800 PMC5439179

[20] Dzudie A., Dzekem B.S. and Kengne A.P. (2017) NT-pro BNP and plasma-soluble ST2 as promising biomarkers for hypertension, hypertensive heart disease and heart failure in sub-Saharan Africa. Cardiovasc. J. Afr. 28, 406–407 29297544 PMC5885052

[21] ancia G., De Backer G., Dominiczak A., Cifkova R., Fagard R., Germano G. et al. (2007) 2007 Guidelines for the Management of Arterial Hypertension: The Task Force for the Management of Arterial Hypertension of the European Society of Hypertension (ESH) and of the European Society of Cardiology (ESC). J. Hypertens. 25, 1105–1187 17563527 10.1097/HJH.0b013e3281fc975a

[22] Huang S., Lv Z., Guo Y., Li L., Zhang Y., Zhou L. et al. (2016) Identification of blood Let-7e-5p as a biomarker for ischemic stroke. PLoS ONE 11, e0163951 10.1371/journal.pone.0163951 27776139 PMC5077157

[23] Dinh Q.N., Drummond G.R., Sobey C.G. and Chrissobolis S. (2014) Roles of inflammation, oxidative stress, and vascular dysfunction in hypertension. Biomed Res. Int. 2014, 406960 10.1155/2014/406960 25136585 PMC4124649

[24] Cayrol C. and Girard J.P. (2018) Interleukin-33 (IL-33): a nuclear cytokine from the IL-1 family. Immunol. Rev. 281, 154–168 10.1111/imr.12619 29247993

[25] De la Fuente M., MacDonald T.T. and Hermoso M.A. (2015) The IL-33/ST2 axis: role in health and disease. Cytokine Growth Factor Rev. 26, 615–623 10.1016/j.cytogfr.2015.07.017 26271893

[26] Griesenauer B. and Paczesny S. (2017) The ST2/IL-33 axis in immune cells during inflammatory diseases. Front. Immunol. 8, 475 10.3389/fimmu.2017.00475 28484466 PMC5402045

[27] Lingel A., Weiss T.M., Niebuhr M., Pan B., Appleton B.A., Wiesmann C. et al. (2009) Structure of IL-33 and its interaction with the ST2 and IL-1RAcP receptors–insight into heterotrimeric IL-1 signaling complexes. Structure 17, 1398–1410 10.1016/j.str.2009.08.009 19836339 PMC2766095

[28] Ho J.E., Larson M.G., Ghorbani A., Cheng S., Vasan R.S., Wang T.J. et al. (2013) Soluble ST2 predicts elevated SBP in the community. J. Hypertens. 31, 1431–1436, 10.1097/HJH.0b013e3283611bdf 23615326 PMC3986262

[29] Yin X., Cao H., Wei Y. and Li H.H. (2019) Alteration of the IL-33-sST2 pathway in hypertensive patients and a mouse model. Hypertens. Res. 42, 1664–1671 10.1038/s41440-019-0291-x 31235844 PMC8075887

[30] Gackowska L., Michałkiewicz J., Niemirska A., Helmin-Basa A., Kłosowski M., Kubiszewska I. et al. (2018) Loss of CD31 receptor in CD4+ and CD8+ T-cell subsets in children with primary hypertension is associated with hypertension severity and hypertensive target organ damage. J. Hypertens. 36, 2148–2156 10.1097/HJH.0000000000001811 29965884

[31] Rudemiller N., Lund H., Jacob H.J., Geurts A.M., Mattson D.L. and PhysGen Knockout Program (2014) CD247 modulates blood pressure by altering T-lymphocyte infiltration in the kidney. Hypertension 63, 559–564 10.1161/HYPERTENSIONAHA.113.02191 24343121 PMC3945169

[32] Touyz R.M. (2008) Transient receptor potential melastatin 6 and 7 channels, magnesium transport, and vascular biology: implications in hypertension. Am. J. Physiol. Heart Circ. Physiol. 294, H1103–H1118 10.1152/ajpheart.00903.2007 18192217

[33] Xue Y.Z., Li Z.J., Liu W.T., Shan J.J., Wang L. and Su Q. (2019) Down-regulation of lncRNA MALAT1 alleviates vascular lesion and vascular remodeling of rats with hypertension. Aging (Albany N.Y.) 11, 5192–5205 10.18632/aging.102113 31343412 PMC6682528

[34] Han X., Wang Q., Wang Y., Hu B., Dong X., Zhang H. et al. (2019) Long non-coding RNA metastasis-associated lung adenocarcinoma transcript 1/microRNA-202-3p/periostin axis modulates invasion and epithelial-mesenchymal transition in human cervical cancer. J. Cell. Physiol. 234, 14170–14180 10.1002/jcp.28113 30633360 PMC13483103

[35] Schmitz B. and Brand S.M. (2016) Uric acid and essential hypertension: the endothelial connection. J. Hypertens. 34, 2138–2139 10.1097/HJH.0000000000001109 27681244

[36] Rodrigo R., Passalacqua W., Araya J., Orellana M. and Rivera G. (2003) Homocysteine and essential hypertension. J. Clin. Pharmacol. 43, 1299–1306 10.1177/0091270003258190 14615465

[37] Wainwright E.N., Jorgensen J.S., Kim Y., Truong V., Bagheri-Fam S., Davidson T. et al. (2013) SOX9 regulates microRNA miR-202-5p/3p expression during mouse testis differentiation. Biol. Reprod. 89, 34 10.1095/biolreprod.113.110155 23843232

[38] Bourckhardt G.F., Cecchini M.S., Ammar D., Kobus-Bianchini K., Müller Y.M. and Nazari E.M. (2015) Effects of homocysteine on mesenchymal cell proliferation and differentiation during chondrogenesis on limb development. J. Appl. Toxicol. 35, 1390–1397 10.1002/jat.3111 25619733

[39] Song Z., Zhao Y., Wang X. and Xu M.J. (2016) Secondary hyperuricemia in chronic renal failure promotes vascular calcification in rats. Sheng Li Xue Bao 68, 709–715 28004064

[40] Yang C., Yao C., Tian R., Zhu Z., Zhao L., Li P. et al. (2019) miR-202-3p regulates Sertoli cell proliferation, synthesis function, and apoptosis by targeting LRP6 and Cyclin D1 of Wnt/β-catenin signaling. Mol. Ther. Nucleic Acids 14, 1–19 10.1016/j.omtn.2018.10.012 30513418 PMC6280020

